# Obesity and the increased risk for COVID-19: mechanisms and nutritional management

**DOI:** 10.1017/S095442242000027X

**Published:** 2020-11-13

**Authors:** Ana Heloneida de Araújo Morais, Thais Sousa Passos, Sancha Helena de Lima Vale, Juliana Kelly da Silva Maia, Bruna Leal Lima Maciel

**Affiliations:** 1 Nutrition Postgraduate Program, Centre for Health Sciences, Federal University of Rio Grande do Norte, Natal, RN 59078-970, Brazil; 2 Biochemistry Postgraduate Program, Biosciences Centre, Federal University of Rio Grande do Norte, Natal, RN 59078-970, Brazil; 3 Department of Nutrition, Centre for Health Sciences, Federal University of Rio Grande do Norte, Natal, RN 59078-970, Brazil

**Keywords:** SARS-CoV-2, Coronavirus, Viral infection, Immunity, Nutritional status, Overweight, ACE, angiotensin-converting enzyme, CCL, chemokine (C-C motif) ligand, COVID-19, coronavirus disease 2019, DIO, diet-induced obesity, ICU, intensive care unit, M1, classical macrophages, PAI-1, plasminogen activator inhibitor-1, SARS-CoV-2, severe acute respiratory syndrome coronavirus 2

## Abstract

The global COVID-19 (coronavirus disease 2019) pandemic has become a complex problem that overlaps with a growing public health problem, obesity. Obesity alters different components of the innate and adaptive immune responses, creating a chronic and low-grade state of inflammation. Nutritional status is closely related to a better or worse prognosis of viral infections. Excess weight has been recognised as a risk factor for COVID-19 complications. In addition to the direct risk, obesity triggers other diseases such as diabetes and hypertension, increasing the risk of severe COVID-19. The present review explains the diets that induce obesity and the importance of different foods in this process. We also review tissue disruption in obesity, leading to impaired immune responses and the possible mechanisms by which obesity and its co-morbidities increase COVID-19 morbidity and mortality. Nutritional strategies that support the immune system in patients with obesity and with COVID-19 are also discussed in light of the available data, considering the severity of the infection. The discussions held may contribute to combating this global emergency and planning specific public health policy.

## Introduction

The global pandemic of COVID-19 (coronavirus disease 2019) caused by the SARS-CoV-2 (severe acute respiratory syndrome coronavirus 2) virus is a complex problem affecting society in several aspects, whether economic, social and especially health. There is no doubt that food plays a fundamental role in the population’s health–disease processes in general: the impact of nutrition on the immune system is well recognised^(1)^.

Nutritional status is closely related to a better or worse prognosis of a disease, and this finding is no different for viral infections^(2)^, including COVID-19. Various nutrients, whether essential vitamins or minerals and the bioactive compounds found in the most diverse foods, especially in fresh or minimally processed foods, play crucial and complementary roles in supporting the immune system’s innate and adaptive processes^(3,4)^. However, the global pattern of food consumption in recent years has been predominantly represented by diets rich in sugars, fats and Na, from ultra-processed industrialised foods^(5-7)^. Despite this, few studies have related the consumption of ultra-processed foods and their respective damage to the immune system^(8,9)^.

According to the FAO the nature of foods, how their choice is driven, and their respective impacts on human health status have been neglected in epidemiological and experimental studies on diet, nutrition and health. However, these preferences have an impact on nutritional status, partially justifying the progressive and sustained increase in obesity cases^(10)^.

Recently, regional boards of the four UN agencies (FAO, UNICEF, WHO and World Food Programme) issued a joint statement on nutrition in the context of the COVID-19 pandemic in Asia and the Pacific. This declaration addresses recommendations for the possible and real impacts of COVID-19 on nutrition, highlighting, among other aspects, the importance of breast-feeding and healthy diets^(11)^.

Obesity has recently been shown to increase the risk for COVID-19 complications, as well as heart disease, diabetes and lung disease^(12,13)^. What puts obesity as a critical problem is that obesity contributes to systemic metabolic dysfunction and is associated with conditions that increase COVID-19 severity. Individuals with obesity also present with changes in the innate and adaptive immune responses, inducing chronic and low-grade inflammation^(14,15)^.

Coronaviruses induce an increase in T-helper 1 (Th1) cells, cytokines (IL-1, IL-6), and inflammatory chemokines (chemokine (C-C motif) ligand 2 (CCL2) protein and C-X-C motif chemokine ligand 10 (CXCL10) protein)^(16-18)^. In this sense, there is a need for additional strategies to support the immune system, especially acquired immunity, to reduce the impact of respiratory and other infections.

Considering these immune dysfunctions in patients with COVID-19, management therapies have used immunomodulatory drugs that benefit COVID-19 patients in pathways also altered by obesity^(19,20)^. Hoffmann *et al.*^(21)^, demonstrated that SARS-CoV-2 uses the SARS-CoV angiotensin-converting enzyme (ACE) 2 receptor to enter the cells and transmembrane protease serine 2 (TMPRSS2) for spike (S) protein priming. According to the study conducted by Sanders *et al.*^(20)^, some adjuvant therapies on COVID-19 deserve special mention, such as the use of corticosteroids and anti-cytokines. A new mechanism related to the inhibition of a cellular protease FURIN, the transmembrane protease, serine 2, encoded by the *TMPRSS2* gene, is an additional target drug for future research.

Because of the little evidence published so far of efficient vaccines for prevention, and specific and effective drugs to treat COVID-19^(20)^, challenges arise, such as obesity being among risk factors with significant implications for COVID-19 mortality. These problems are likely to overload health systems that are mostly not well configured to manage patients with obesity^(22,23)^.

Thus, this review presents the health implications of obesity considering the COVID-19 pandemic. We explain diets inducing obesity, and the importance of the different kinds of foods in this process. We review tissue disruption in obesity, leading to impaired immune responses and the possible mechanisms by which obesity and its co-morbidities increase COVID-19 morbidity and mortality. Nutritional strategies that support the immune system in patients with obesity and with COVID-19 are also discussed in light of the available data, considering the severity of the infection for nutritional therapy. The discussions held may contribute to combating this global health emergency, and help the planning of specific public health policy.

## Different aspects related to obesity and the COVID-19 pandemic

Until 2020, obesity was the main pandemic of the 21st century^(24-26)^. Nevertheless, obesity has never received urgent attention, as occurs with the rapid spreading of infectious diseases. Now, the world faces the confluence of two overlapping public health problems: COVID-19 and obesity – with its associated co-morbidities^(22)^.

According to the WHO^(27)^, global obesity has almost tripled since 1975. More alarming data are that, in 2018, 40 million children under 5 years of age were overweight or obese. The USA, the country at the top of the COVID-19 pandemic ranking, is also one of the leaders in the prevalence of obesity^(28)^.

Despite the alarming numbers of overweight and obesity cases, the WHO^(27)^ considers obesity to be preventable. Classic interventions, with the highest degree of therapeutic recommendation, consist of practising regular physical activity, important in the anti-inflammatory response and reduction of changes in cytokines associated with the strengthening of cellular immune function in obesity^(29)^. Limiting the intake of energy from fats, reducing salt and sugars, and increasing the consumption of vegetables, fruits and whole grains is also essential^(27)^. However, these measures are not easy to adhere to, considering that patients with obesity and its associated morbidities keep increasing in prevalence^(30,31)^.

With the ongoing COVID-19 pandemic, these recommendations have been reinforced by relevant agencies of the UN, which consider that a balanced, diversified and nutritious diet is a crucial way of promoting health and nutritional well-being^(11)^. This nutritional care plays a critical role in the immune system^(3,4)^.

Altered microbiota diversity occurs in most individuals with obesity, and a mutual interplay exists between gut microbiota and the immune system^(32)^. According to Morais *et al.*^(33)^, the administration of probiotics could be a promising adjuvant alternative in the treatment of SARS-CoV-2 infection in addition to adequate intake of nutrients and bioactive compounds from unprocessed food.

Inadequate intake of vitamins and minerals, food processing and industrialisation are related to obesity and the co-regulating of the intestinal microbial ecosystems. Increased energy intake, influenced by lifestyle changes resulting from urbanisation and globalisation, has promoted an obesogenic environment^(32,34-38)^. Although consuming ultra-processed foods is associated with obesity and its co-morbidities^(39)^, only recently studies related this consumption to damage in the immune system^(13,14)^. Cohort studies are also being conducted to clarify the real impact of ultra-processed foods on health^(40,41)^.

Thus, the viability of widespread interventions related to this dietary pattern and their sustainability needs to be evaluated, considering the possibility of generating economic and social impact^(30,42)^. These new interventions should add to the continued adoption of recommendations and strategies aligned with the guidelines given by competent entities in generating data and managing aspects related to nutritional surveillance, food protection, quality of food and food security. These concerns have been raised given COVID-19^(11)^, as food biodiversity is an opportunity to increase food and nutrition security today^(43)^.

Multisectoral and evidence-based policy actions to deal with the global nutrition crisis have risen. Consequently, obesity has been targeted in several aspects, even though some measures are still unpopular and do not present immediate results. Actions in health systems, economic incentives, concerns about the school and work environment, quality and labelling standards, and innovation, entrepreneurship and sustainability are being implemented^(44-47)^.

Overweight and obesity cause other non-communicable diseases that affect the reserves of many nutrients^(48-52)^. Overweight and obesity lead to adverse metabolic effects on blood pressure, lipid profile, insulin resistance, low-grade inflammation, and, consequently worse prognosis for viral infections, and this seems to be the same for SARS-CoV-2^(2,12,13,15)^.

However, most deaths from non-communicable diseases could be prevented with known behavioural and pharmaceutical interventions. Several initiatives for preventing and controlling non-communicable diseases have been adopted over the last decades, supported by the WHO. These initiatives are currently being tested in the treatment of COVID-19, especially in cases where obesity is also present^(15,19,20)^.

Besides obesity emerging as a significant important risk factor for the severity of COVID-19, the lockdown imposed by COVID-19 may also increase the prevalence of obesity^(53)^. For example, most weight-loss programmes and nutritional interventions, such as bariatric surgery, are currently restricted. Additionally, self-isolation measures adopted in some countries will directly affect food consumption at this time of anxiety, and restrict mobility and physical activity. Thus, the effects of increased overweight and obesity may persist for an extended period^(54)^, even considering the alternating isolation. This increase may be especially true in vulnerable populations that already presented food and nutritional insecurity as social-health issues and may worsen the evolution of the COVID-19 pandemic even more^(11,55-60)^.

Finally, this global urgency to combat COVID-19 may generate more confidence in processed and industrialised foods compared with fresh food, conditioned by hygienic-sanitary safety, longer shelf life and easier stocking^(61)^. According to Butler & Barrientos^(62)^ and Muscogiuri *et al.*^(63)^, it is essential to improve broader access to healthy foods rich in vitamins, minerals, bioactive compounds and antioxidants to reduce susceptibility and long-term complications of COVID-19. Diet composition and obesity have a widespread role in immunity^(32)^, affecting the severity of respiratory diseases and other infections.

## Obesity and impaired immune response

Impaired immune response in individuals with obesity is not a novel feature, and this has been evidenced by high vaccine failure^(64)^ and more complications from infections^(65)^. Worsened response to infections in the presence of obesity was observed in Asian (1957–1960) and Hong Kong (1968) influenzas, leading to higher mortality and prolonged illness duration. The H1N1 pandemic in 2009 was also more severe in patients with obesity, causing more hospitalisations and deaths^(66)^. The same seems to be true for the COVID-19 pandemic^(23,66)^, and there are some immunological explanations for these facts.

One of these explanations underlies the fact that obesity causes stress and dysfunction in many tissues, including the adipose tissue, liver, skeletal muscle, pancreas, gut and respiratory tract. Most of these alterations present an intricate association with inflammation, determined by the immune cells activated in the adipose tissue^(14)^.

Low-grade chronic inflammation, characterised by a higher concentration, in basal levels, of the pro-inflammatory cytokines TNF-α, IL-1β, monocyte chemoattractant protein-1 (MCP-1) and IL-6, is an obesity hallmark. These cytokines are mainly produced by visceral and subcutaneous adipose tissue-activated macrophages and enlarged adipocytes^(67)^. Classically, this production was considered separately for obesity and pathogens, but now it is accepted that both cause chronic cellular stress, activating shared signalling pathways. The primary difference between these two activations is that the response to pathogens is acute and highly inflammatory. In obesity, the low-grade tissue inflammation provides a physiological immune adaptation. Disruption of this adaptation causes the transition from metabolically healthy obesity to the metabolic syndrome^(68)^.

Although the order of the determining factors for obesity-induced inflammation in humans is not precisely described, animal models have shown that overfeeding causes oxidative stress and an increase in NEFA in the adipose tissue^(68)^. These factors induce the activation of classical macrophages (M1), which are highly inflammatory cells, producing IL-1β, TNF-α and IL-6. TNF-α reduces insulin sensitivity, and the inflammatory cytokines induce more lipolysis with the release of more NEFA, creating an upstream pathway for the inflammation^(69)^.

The enlarging white adipose tissues, in turn, release the CCL2, CCL5 and CCL8 chemokines, which recruit more inflammatory monocytes to the adipose tissue. Concisely, in the absence of obesity, adipose tissue macrophages represent 10–15 % of stromal cells and present markers associated with the maintenance of insulin sensitivity by the production of IL-10. In this context, alternative macrophages (M2) are more activated, producing IL-10 after stimulation by IL-4 and IL-13. Adiponectin is also higher in lean animals and humans and inhibits the activation of M1. In contrast, during obesity in mice and humans, inflammatory monocytes are recruited to adipose tissue, increasing macrophage content to 45–60 %^(14,69)^.

The inflammation and stress in the adipose tissue induce adipocyte apoptosis and the release of chemotactic mediators. Apoptosis and the chemotactic mediators promote inflammatory leucocyte infiltration. The inflammatory leucocytes produce resistin and IL-1β and induce M1 and adipocytes to produce more TNF-α, IL-6 and monocyte chemoattractant protein-1 (MCP-1), causing a vicious cycle of inflammation^(67-69)^.

These inflammatory alterations in the adipose tissue underlie the chronic systemic inflammation and the metabolic changes in obesity. These changes are mediated, besides insulin resistance, by unfavourable hormone milieu. Low adiponectin (an anti-inflammatory adipokine) and high leptin (pro-inflammatory adipokine) production is observed in obesity, ultimately stimulating NF-κB-mediated pro-inflammatory gene transcription and reducing PPAR-α and PPAR-γ transcriptional activity. This hormone profile also leads to dysregulation of the immune response and may concur with the obesity-linked complications during infections^(70)^.

Obesity also negatively alters the lymphoid tissue structure and function. This impairment occurs by ectopic lipid accumulation in the bone marrow, thymus and secondary lymphoid organs, changing the immune tissue architecture^(71,72)^. This architecture is essential to the development of functional leucocytes through the critical interactions within the immune cells^(14)^. These tissue structure changes alter leucocyte populations’ distributions, lymphocyte activity, and immune defences and are linked to a compromised response of innate lymphoid cells^(73)^.

Interestingly, accumulation of fat tissue in the lymphoid tissues naturally occurs in senescence and impairs immunity in the elderly^(74-76)^. In animal models, energy restriction has been found to block this fat accumulation and is associated with increased immunity and longer lifespan. Thus, obesity can cause premature ‘ageing’ of the immune system^(71,72)^. This fact may be one of the causes putting individuals with obesity at risk for severe influenza and COVID-19 evolutions, with increased mortality. Further studies should address how immunomodulation induced by diets and other agents could revert the effects of fat accumulation on lymphoid tissues from individuals with obesity and older individuals in the context of SARS-CoV-2 infection.

Obesity alters gut microbiota^(67)^ and several studies have associated oxidative stress with dysbiosis^(77,78)^, which is the disruption of healthy microbiota^(79)^. Evidence suggests that intestinal microflora may be responsible for the development of the low-grade inflammation in obesity. This could possibly occur by microflora dysfunctions in the intestinal barrier, increasing its permeability and inducing endotoxaemia, characterised by an increase in gut-derived plasma lipopolysaccharide^(80,81)^.

Studies in animal models have shown that communication between the gut, adipose tissue and brain is essential for maintaining energy balance. This communication is impaired during obesity and type 2 diabetes^(82)^. In this context, metabolic endotoxaemia has been identified as one of the main factors leading to the development of metabolic inflammation and insulin resistance because the increase in plasma lipopolysaccharide induces M1^(83)^. The adipokines secreted by the adipose tissue might also affect the airway function. Leptin is involved in neonatal lung development, surfactant production^(84,85)^ and regulation of ventilatory drive^(84,86)^. Studies have consistently shown the association of high leptin concentrations and asthma^(87,88)^.

Considering respiratory infections, most of the knowledge linking obesity and immunity comes from experimental models of influenza^(89)^. In diet-induced obesity (DIO) mice with influenza A infection, impaired host defence was associated with a decrease in type I interferon (IFN-α and IFN-β), a delay in IL-6 and TNF-α expression, that increased to higher concentrations than those in lean animals, and impaired the cytotoxicity of natural killer cells^(90)^. Further studies also revealed that, after influenza infection, DIO reduced dendritic cells’ ability to present antigens to T cells, impairing monocyte and CD8^+^ T cell recruitment and reducing IL-2 and IL-12 production^(91)^. DIO mice also demonstrated an inability to produce and maintain functional antigen-specific memory CD8^+^ T cells for influenza viruses^(92,93)^. Additionally, effector CD8^+^ T cells presented a lower ability to kill influenza-infected cells, and compromised healing of pulmonary epithelial cells, resulting in microvascular permeability and protein leak^(89)^.

**Fig. 1.**
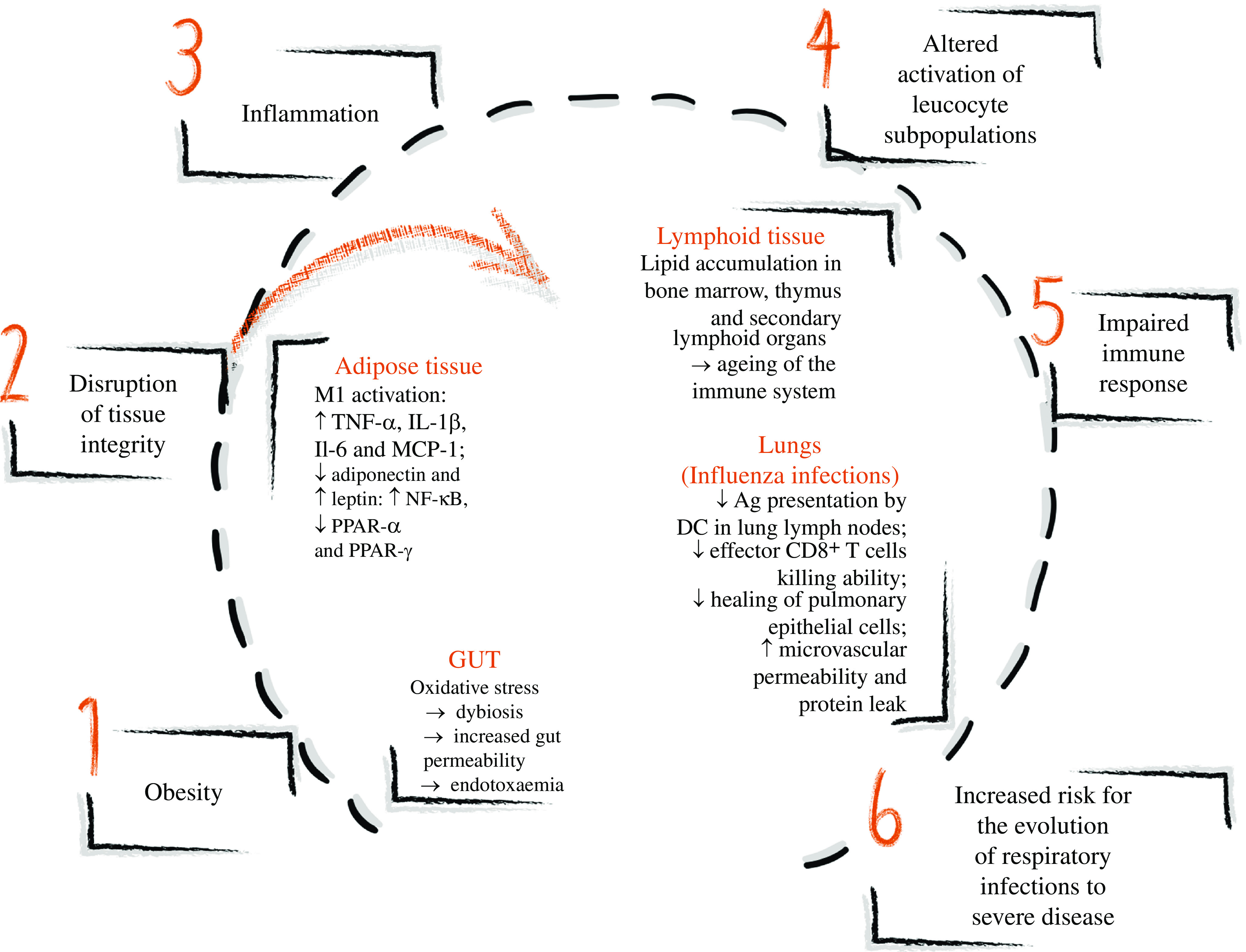
Obesity and immune alterations in different tissues leading to impaired immune response and increased risk for the evolution of respiratory infectious to severe disease. Obesity determines the stress and disruption of many tissues’ integrity leading to inflammation. In the adipose tissue, the enlarging adipocytes present oxidative stress and increase the release of NEFA in the adipose tissue, activating classical macrophages (M1), which produce IL-1β, TNF-α and IL-6. An unfavourable hormone milieu also promotes inflammation: low adiponectin and high leptin productions are observed in obesity. In the lymphoid tissue, lipid accumulation occurs in the bone marrow, thymus and secondary lymphoid organs, altering the immune tissue architecture similarly to findings observed in ageing. In the gut, oxidative stress causes dysbiosis, increasing gut permeability and inducing endotoxaemia, characterised by an increase in gut-derived plasma lipopolysaccharide, which induces inflammation. In the lungs, experimental diet-induced obesity studies of influenza infections showed the reduced ability of dendritic cells (DC) to present antigens (Ag) to T cells, impairing monocyte and CD8^+^ T cell recruitment and reducing IL-2 and IL-12 production. Effector CD8^+^ T cells demonstrated a lower ability to kill influenza-infected cells, and the healing of pulmonary epithelial cells is compromised, resulting in microvascular permeability and protein leak. Overall, the inflammation induced by obesity causes altered activation of leucocyte subpopulations, impairing the immune response and increasing the risk of the evolution of respiratory infection to severe disease. ↑, Increase; ↓, decrease.

Thus, obesity causes stress and disruption of several tissues, with inflammation mainly in the adipose and lymphoid tissues, gut and respiratory tract. The stress and inflammation determine the altered activation of leucocyte subpopulations. These changes impair the immune response, increasing the risk for the evolution of infections to severe disease, and might be the leading causes for increased mortality in patients with obesity and COVID-19 (Fig. 1).

## Obesity, co-morbidities and COVID-19: a triple burden

In addition to disruption of the immune system, obesity can result in metabolic dysfunction leading to dyslipidaemia, insulin resistance, type 2 diabetes, hypertension and CVD, all of which increase patients’ vulnerability to SARS-CoV-2 infection^(94)^. Thus, obesity plays a crucial role in the pathogenesis of COVID-19.

Dietz & Santos-Burgoa^(12)^ suggested that the increased prevalence of obesity in older Italian adults compared with in Chinese could explain the higher COVID-19 mortality in Italy. Studies in both animals and human subjects have shown that obesity is directly related to more severe respiratory infections^(89)^. The H1N1 influenza pandemic (2009) reinforced this evidence since multiple cohort studies showed that obesity and morbid obesity are independent risk factors that increase hospitalisation, admission and time in an intensive care unit (ICU)^(95,96)^. In Spain, a study observed that obesity was the most common co-morbidity (48 %) in patients admitted to ICU with SARS-CoV-2 infection^(97)^.

Although all the possible pathways of interaction between COVID-19 and obesity are still not wholly understood, the discussion concerning the impact of the high prevalence of obesity and coronavirus infection has arisen. Possibly, the interaction between these two important public health concerns occurs through multiple overlapping pathways, some of which will be discussed in this section. Data on this topic are still limiting, and for now, only a few studies have addressed the triple burden of obesity, its co-morbidities and COVID-19 or even other coronavirus diseases. Those data are summarised in Table 1.

**Table 1.** Studies concerning co-morbidities of obesity and coronavirus infections

Reference	Study design	Co-morbidity associated with obesity	Relationship with the worsening prognostic of coronavirus infections
Yang *et al.*^(164)^	Human study, retrospective analysis	Hyperglycaemia and hypertension	Patients admitted to Beijing hospitals diagnosed with SARS showed increased risk of severe hypoxia and death risk, due to hyperglycaemia and ketosis effects
Kulcsar *et al.*^(105)^	Mouse model	Type 2 diabetes	Mice with diabetes showed increased expression of IL-17a after infection by MERS-CoV
Al Heialy *et al.*^(98)^	Re-analysis of publicly available transcriptomic data and mouse model	Diabetes	Human pulmonary epithelial cells infected with SARS-CoV-2 presented positive regulation of the suppressor of the *SOC3* gene, which regulates inflammation and inhibits leptin signalling, favouring viral replication. A mouse model of diet-induced obesity showed increased ACE2 expression in the lungs by the suppression of genes that encode SREBP1
Zheng *et al.*^(118)^	Human case–control study	MAFLD	A multi-centre study involving 214 patients with laboratory-confirmed COVID-19 aged between 18 and 75 years of three hospitals in the city of Wenzhou (China) to investigate the association between MAFLD and COVID-19 severity. Data showed that the presence of obesity in patients with MAFLD increased six-fold the risk for severe COVID-19

SARS, severe acute respiratory syndrome; MERS-CoV, Middle East respiratory syndrome coronavirus; SARS-CoV-2, severe acute respiratory syndrome coronavirus 2; *SOC3*, suppressor of cytokine signalling 3; ACE2, angiotensin-converting enzyme 2; SREBP1, sterol regulatory element-binding protein 1; MAFLD, metabolic associated fatty liver disease; COVID-19, coronavirus disease 2019.

Patients with obesity can present compromised pulmonary ventilation dynamics because overweight increases pressure in the diaphragm, making breathing more difficult during infection, promoting a relative increase in the anatomical dead space. Thus, decreased expiratory reserve volume, functional capacity and respiratory system compliance occur^(12,66,98)^. In general, abdominal obesity can impair the ventilation in the lungs’ base, resulting in reduced oxygen saturation of the blood^(99)^ and obstructive sleep apnoea associated with an increased risk of CVD and metabolic disease^(100)^. This problem can contribute to daytime alveolar hypoventilation (obesity hypoventilation syndrome) and apparent respiratory failure^(101)^.

Obesity can also contribute to the development of more virulent diseases, and permissive mutations might be triggered by delayed interferon responses that favour viral replication^(102)^. Meliopoulos *et al.*^(103)^, using an obese mouse model, showed that the lungs of obese mice increased the expression of epithelial integrin 6, which was associated with the severity of lung disease in an influenza virus infection.

Patients with hyperglycaemia and type 2 diabetes have a state of metabolic inflammation, promoting the release of inflammatory cytokines^(104)^. In cases of COVID-19, this condition might be one of the triggering factors to the so-called cytokine storm (a high cytokine release), consequently leading to death due to multiple organ failure. Kulcsar *et al.*^(105)^ (Table 1), using mice with type 2 diabetes induced by administering a high-fat diet, observed that upon infection with MERS-CoV (Middle East respiratory syndrome coronavirus), diabetic mice had a prolonged phase of severe disease and delayed recovery, regardless of virus titres. These results were attributed to a dysregulated immune response, characterised by lower TNF-α, IL-6, IL-12b and Arg1 expression levels and higher IL-17a expression levels.

Recently, Hoffmann *et al.*^(21)^ elucidated that SARS-CoV-2 enters human cells through spike glycoprotein found on the virus’s surface. This spike is capable of binding to ACE2, which is expressed in the mouth^(106)^, lung^(21)^, intestine^(107)^ and kidney^(108)^. Studies have already shown that the renin–angiotensin system modulates the metabolism and endocrine function of adipocytes^(109)^, so this will have significant consequences in patients with obesity infected with SARS-CoV-2.

Bornstein *et al.*^(15)^ discuss that individuals with insulin resistance or diabetes and hypertension may have this clinical condition aggravated due to an imbalance in the pathways that regulate the activities of ACE1 and ACE2. An increase in the activation of angiotensin receptors (AT1R and AT2R) is promoted, causing higher pro-inflammatory responses, and stimulating aldosterone secretion. As a result, there is an increment in local vascular permeability, which worsens the respiratory syndrome. Besides, type 2 diabetes induces the expression of ACE in tissues other than the lung, such as the liver and heart, so this may contribute to the death of patients due to multiple organ failure in cases of SARS-CoV-2. They observed a high risk to the development of severe hypoxia and death related to the hyperglycaemia and ketosis effects in severe acute respiratory syndrome (SARS) (Table 1).

The clinical condition of patients diagnosed with SARS-CoV-2 worsens more intensely in a pre-existing CVD, related to increased ACE2 secretion^(110)^. As reviewed by Tan & Aboulhosn^(111)^, several studies have shown that patients with CVD (hypertension and coronary artery disease) require considerable attention in the ICU due to the higher possibility of developing a severe clinical condition of COVID-19.

Obesity and diabetes are directly related to unregulated lipid synthesis and clearance. Al Heialy *et al.*^(98)^, through the re-analysis of public available transcriptomic data, showed that unregulated lipogenesis leads to high expression of ACE2 in individuals with obesity. This mechanism is consequently related to worse lung injury in SARS-CoV-2 infections (Table 1).

Moreover, abdominal fat is associated with metabolic risk factors that contribute to increased coronary risk. Among these factors are abnormalities in the coagulation system^(112)^. According to the review by Blokhin & Lentz^(113)^, fibrinolysis is the process of degradation of the fibrin clot by plasmin, whose rate is down-regulated by a serine protease inhibitor secreted by the vascular endothelium, liver and adipose tissue called plasminogen activator inhibitor-1 (PAI-1). The expression of the inhibitor undergoes positive regulation in visceral adipose tissue in obesity. According to the review by Blokhin & Lentz^(113)^, high plasma levels of PAI-1 are observed in patients with obesity or the metabolic syndrome. Studies show that TNF-α positively regulates the expression of PAI-1, which suggests that high levels of this activator are related to the chronic inflammatory state of obesity. Preclinical studies demonstrate the association between obesity, high levels of PAI-1, and thrombosis, which reinforces that PAI-1 plays a crucial role in promoting the prothrombotic effects of obesity.

There is an increased risk of disseminated intravascular coagulation and venous thromboembolism as complications in COVID-19^(114)^, and this may be even worse in patients with obesity. Shock and disseminated intravascular coagulation are associated causes of organ dysfunction in sepsis, and abnormal coagulation has been found in non-survivors of COVID-19. Yin *et al.*^(115)^ observed that the platelet count in patients with COVID-19 was significantly higher than in the non-COVID group. They attributed this increase to the reactive increase in thrombopoietin after the lung infection, characterising more severe inflammatory reaction and hypercoagulability in COVID-19.

Patients with acute coronary syndrome deserve greater care by specialist clinicians because cardiac functional reserve may be compromised due to myocardial ischaemia or necrosis. When infected with SARS-CoV-2, these patients may have a myocardial infarction (type 1), increased myocardial demand, ischaemia and necrosis, leading to myocardial infarction type 2. They may also have an increase in the metabolic demand that leads to heart failure and death^(110)^.

According to Kovesdy *et al.*^(116)^, metabolic changes in obesity might also affect the kidneys through the induction of hypertension and type 2 diabetes. Numerous renal system changes can occur, including ectopic lipid accumulation, increased fat deposition in the renal site, development of glomerular hypertension, increased glomerular permeability due to damage to the glomerular filtration barrier related to hyperfiltration, development of glomerulomegaly, and focal or segmental glomerulosclerosis. Insulin resistance and obesity are also related to the increased risk of developing nephrolithiasis. Body weight is associated with lower urinary pH and increased urinary oxalate, excretion of acids, Na^+^ and phosphate. Thus, patients with obesity-associated kidney complications may be more severely affected by SARS-CoV-2 infections. Xu *et al.*^(117)^ were able to locate the expression and distribution of the *ACE2* and *TMPRSS* genes in the renal cells of patients diagnosed with COVID-19, through single-cell transcriptome sequencing (scRNA) analysis. They found that the podocytes and cells of the proximal straight tubules were infected by SARS-CoV-2, which resulted in chronic kidney failure induced by the virus.

Zheng *et al.*^(118)^ highlight that patients with metabolic associated non-alcoholic fatty liver disease generally have obesity. As a result, they have metabolic risk factors that may generate a higher risk of respiratory illnesses. Consequently, this can contribute to increased severity of SAR-CoV-2 infection (Table 1).

**Fig. 2.**
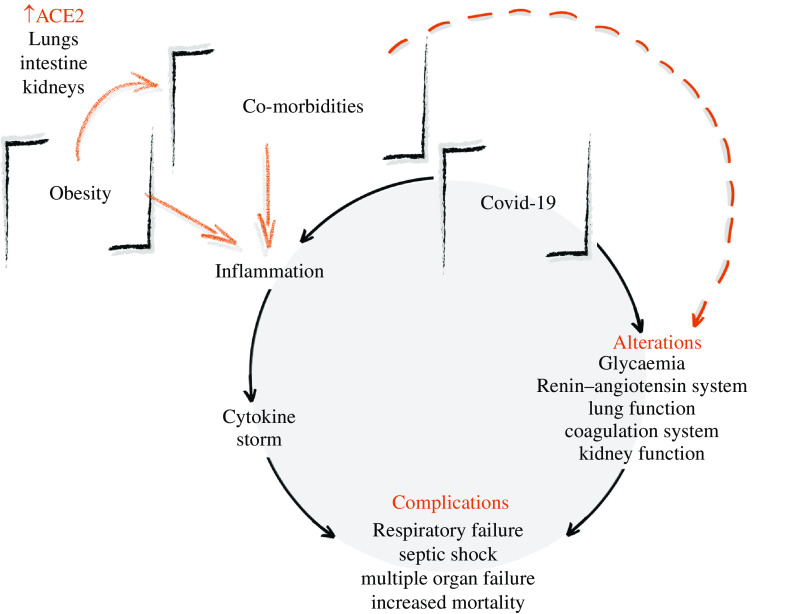
Obesity, co-morbidities and COVID-19 (coronavirus disease 2019): the triple burden. Obesity and its co-morbidities induce low-grade chronic inflammation and an increase in angiotensin-converting enzyme 2 (ACE2) receptors present in the lungs, intestine and kidneys. COVID-19, in turn, also induces inflammation and alterations overlapping those induced by obesity and its co-morbidities. These alterations occur as the disease severity increases and are in: glycaemia – increasing blood glucose; the renin–angiotensin system – causing higher pro-inflammatory responses; lung function – increasing vascular permeability and lung injury; the coagulation system – increasing prothrombotic effects; kidney function – inducing chronic kidney failure. Possible pathways to explain these worsened alterations in patients with obesity and with co-morbidities are: in the lungs – a higher ACE2 expression, unregulated lipogenesis and inflammation; in the coagulation system – a higher concentration of plasminogen activator inhibitor-1; and in the kidney – a higher expression of *ACE2* and transmembrane protease serine (*TMPRSS*) genes. These overlapping alterations and inflammation associated with the cytokine storm induced by COVID-19 increase the risk of complications in patients with obesity, such as respiratory failure, septic shock, multiple organ failure, and, ultimately, increased mortality.

Thus, obesity and its low-grade chronic inflammation induce the onset of the most diverse co-morbidities. Obesity and its co-morbidities, in turn, increase the risk for severe COVID-19. The SARS-CoV-2 infection itself might provoke a broad spectrum of inflammatory responses, aggravating the inflammation of obesity and its co-morbidities and inducing the cytokine storm, characterising the triple burden between obesity, co-morbidities, and COVID-19 that increases mortality (Fig. 2).

## Impact of COVID-19 on the nutritional management of obesity

The first symptoms of COVID-19, such as cough, myalgia, fever and shortness of breath, are similar to other common viral infections^(119)^ and, in general, individuals can be recovering at home. However, patients with obesity should be aware that they may have more complicated clinical courses, as previously discussed.

When presenting with fever, even if not thirsty, COVID-19 patients should continue drinking fluids to support the body’s ability to fight the virus and the immune function. The American Society for Parenteral and Enteral Nutrition (ASPEN)^(120)^ recommends drinking water or clear liquid fluids every hour, at least 3 litres/d, and to monitor for signs of dehydration during COVID-19. There have been reports of olfactory and gustatory dysfunctions, like anosmia and ageusia, in patients with mild-to-moderate SARS-CoV-2 infection^(121-123)^. These symptoms can underlie the loss of appetite, also related as a symptom of COVID-19.

Loss of appetite is a symptom of COVID-19^(123)^ and has already been linked to inflammation^(124)^. Regardless of age or an individual’s BMI, people with a loss of appetite decrease energy and micronutrient consumption^(125,126)^. Nutrients have been extensively related to improvement of the immune status and, consequently, the response to COVID-19. Fresh and minimally processed foods, sources of vitamins C, A, D and E, B vitamins, *n*-3 PUFA, Se, Fe and Zn should be encouraged^(4,127)^. The dietary references intake values of these vitamins and minerals were proposed for healthy individuals by the Institute of Medicine and should be met by the consumption of these foods^(128-131)^.

Evidence suggests that obesity may decrease fat-soluble vitamins and vitamin C concentrations in the plasma^(50,132,133)^. For vitamin A or β-carotene (vitamin A precursor), this reduction occurs due to a lower intake of these nutrients and/or higher deposition in the adipose tissue, decreasing its bioavailability^(134,135)^.

Vitamin A deficiency has been associated with lower vitamin C concentrations. In a group of 191 patients with the metabolic syndrome, vitamin C deficiency increased 3·5-fold the risk of vitamin A deficiency^(50)^. Vitamin A (or its precursors) can be provided by a large amount of fresh or minimally processed foods, such as milk and dairy products, vegetables, fruits (for example, cantaloupe melon), eggs, and oils^(47,136)^. Males have daily requirements slightly higher than females^(129)^. Citric fruits and dark green vegetables are the main sources of ascorbate in the human diet. One portion of orange, kiwi, guava, cantaloupe melon, broccoli or Brussels sprouts has a minimum of 50 % of the RDA of vitamin C^(131,137)^.

A major source of vitamin D for most humans comes from exposure of the skin to sunlight. Thus, exposure of arms and legs to 0·5 minimal erythemal doses is equivalent to ingesting approximately 3000 IU (75 μg) of vitamin D_3_. Some foods naturally contain vitamin D_2_ or vitamin D_3_ (cold water fishes, like sardines, tuna, salmon; egg yolk; shitake mushrooms) and fortified food (milk, butter, cheese, yogurts, breakfast cereal). Pharmaceutical and supplemental sources have different contents of vitamin D_2_ or D_3_^(138)^.

The Endocrine Society Clinical Practice recognises that obesity is associated with vitamin D deficiency and suggests that the daily requirements are higher than the Institute of Medicine values^(128)^, ranging from 1500 to 2000 IU/d (38 to 50 μg/d) and upper-level intakes until 10 000 IU/d (250 μg/d)^(138)^. Vitamin D supplementation to raise serum 25-hydroxyvitamin D (25(OH)D) concentrations at least 40–50 ng/ml (100–125 nmol/l) would be an important step in preventing COVID-19 infection and spread^(139)^.

The relationship between Zn, obesity and diabetes is well described, showing how this trace element influences lipid and insulin profiles, adiposity, inflammatory processes and insulin resistance^(140-143)^. Recent studies have shown that overweight people and individuals with obesity had lower Zn serum levels than normal-weight individuals^(142,144)^. These lower Zn levels in biological fluids have been associated with a higher risk of developing insulin resistance, inflammation, hypertension and hypertriacylglycerolaemia^(145)^. Another study revealed that an energy-restricted diet with 30 mg/d of Zn reduced inflammatory markers, insulin resistance, anthropometric measurements and appetite in individuals with obesity^(146)^.

Zn is in many foods, including meat, fish, oysters, nuts and legumes. Although the absorption varies by substrate, this trace element plays a role as an immunonutrient together with vitamins A, C, E and D, and Se and Fe^(147,148)^. Besides, high Zn^2+^ concentrations can mitigate viral RNA replication. A study showed that the replication of RNA-dependent RNA polymerase (RdRp) from coronavirus (SARS-CoV nsp12) was inhibited by increased Zn^2+^ concentrations^(149)^.

Dysbiosis has been observed in patients infected with COVID-19. Some experiences in managing this alteration have reported the administration of prebiotics and probiotics^(150)^. There is a mutual interaction between obesity, gut microbiota and the adaptive immune system^(32,67)^. These interactions possibly increase the inflammatory status in patients with viral infections, as discussed by Gogokhia *et al.*^(151)^ in patients with HIV.

Other symptoms of COVID-19 that contribute to the maintenance of overweight and obesity are severe fatigue or myalgia. These can lead to decreased physical activity^(152,153)^. Physical inactivity leads not only to weight gain but also to a loss in immune competence^(154,155)^. Staying home made it more challenging to maintain motivation to a physical activity schedule. For those patients with obesity following nutritional counselling and using medication for co-morbidities and other conditions related to obesity, purchasing healthy food and medicines might be difficult, especially during a lockdown.

The period of social distancing imposed in most countries can be particularly challenging for individuals in treatment for obesity^(152,155)^. For these cases, it is essential to maintain a stock of medicines and groceries^(155)^, and have a fresh food delivery service, so that these items can be replaced more frequently. The amount of contact and support from clinical visits influences motivation to maintain lifestyle changes for weight loss. Teleconference, telehealth and mobile devices may be an effective alternative to provide this patient support and counselling process^(154,156)^. In this social isolation context, sensations that trigger food consumption are also difficult to contain, such as anxiety and depression^(155)^. The boredom caused by the broken routine leads to food craving in an attempt to achieve mood improvement. Therefore, keeping fresh foods and planning meals could help tackle homestay’s adverse health effects^(63)^.

In response to the COVID-19 pandemic outbreak, the International Federation for the Surgery of Obesity and Metabolic Disorders (IFSO) postponed all elective surgical and endoscopic bariatric surgery to keep hospital resources available to COVID-19 coping^(157)^. As mentioned, this can negatively affect individuals since following a low-energy diet and medical weight management can be difficult, causing immeasurable impacts on health^(158-160)^. Stahel^(160)^ classified bariatric surgery as elective discretionary that could be postponed for 3 months. Additionally, this author proposed a decision-making algorithm for utilisation during the current COVID-19 pandemic.

The clinical spectrum of the SARS-CoV-2 infection ranges from mild disease with non-specific signs to symptoms of acute respiratory illness^(119)^. In situations of worsening symptoms, individuals must seek medical attention. Currently, several channels are available such as phone calls, chats, videoconferences, where it is possible to consult and ask questions to specialists^(156)^.

However, sometimes the individual must go to a health service for examinations and even for specific hospitalisation care when diagnosed with COVID-19^(119)^. Hospitalised patients with obesity should be kept on oral feeding whenever possible. Early nutritional supplementation of non-ICU hospitalised patients aims to supply energy and protein that may have its needs increased in situations of infection and inflammation by a coronavirus^(126)^. If oral feeding is not tolerated and/or respiratory conditions require non-invasive ventilation or continuous positive airway pressure, supplemental/total parenteral nutrition may be a choice to avoid complications related to concurrent use of face masks and enteral nutrition^(126)^.

Obesity increases the risk of hospitalisation^(154)^. As previously discussed, in situations of more considerable aggravation, an overreaction of the immune system leading to the lungs’ autoimmune aggression could be involved in the most severe acute distress respiratory syndrome^(119)^. Therefore, it may be necessary to continue treatment in ICU, especially for adequate ventilatory support considering the high pulmonary demand characterising COVID-19.

According to the recent European Society for Parenteral and Enteral Nutrition (ESPEN) guidelines, enteral nutrition may be superior to parenteral nutrition because of a lower risk of infection and is security for patients who receive mechanical ventilation^(154)^. Prone position ventilation is the therapy of choice in acute respiratory distress syndrome patients with obesity^(161)^, and enteral nutrition in this position is not a limitation, being viable and safe.

However, situations such as uncontrolled shock, hypercapnia, acidosis and life-threatening hypoxaemia require a delay in enteral nutrition^(162)^. In cases of impaired enteral nutrition, parenteral nutrition should be suggested. It is vital to consider the target level for blood glucose between 6 and 8 mmol/l, monitoring blood TAG and electrolytes, including phosphate, K^+^ and Mg^2+(154)^.

The best way to determine the energy needs of individuals is indirect calorimetry. If this is not available, Caccialanza *et al.*^(126)^ suggest that the total amount of energy should be estimated by multiplying the resting energy expenditure calculated by the Harris–Benedict equation by a 1·5 stress-factor (in patients with obesity (BMI > 30 kg/m^2^), ideal body weight used in the equation), and 1·3 to 1·5 g of proteins/kg of ideal body weight^(126,154)^.

Another option for energy estimation is to use 11–14 kcal/kg per d (46–59 kJ/kg per d) with actual body weight for critically ill patients with BMI 30–50 kg/m^2^, and 22–25 kcal/kg per d (92–105 kJ/kg per d) with ideal body weight for critically ill patients with BMI > 50 kg/m^2^, as proposed by Mogensen *et al.*^(163)^, when analysing patients with obesity and with super-obesity on mechanical breathing. Schetz *et al.*^(161)^, for energy from non-protein sources, consider an energy ratio from fat and carbohydrates between 30:70 % (subjects with no respiratory deficiency) to 50:50 % (ventilated patients)^(154)^.

Thus, the nutritional management of SARS-CoV-2 infection in patients with obesity, although not fully established yet, must consider the infection’s severity. Several nutrients deserve special attention in immunomodulation, such as vitamins A, D and C, and minerals, such as Zn. Prebiotics and probiotics may also be necessary for COVID-19 management, especially in patients with obesity. With the advances of research in the area, health professionals and researchers should establish protocols to ensure proper care.

## Final considerations

The SARS-Cov-2 infection has become pandemic, overlapping with obesity and its co-morbidities. Obesity and its co-morbidities create an unfavourable inflammatory environment that enhances the risk of severe COVID-19. Therefore, obesity must be recognised as an independent risk factor for COVID-19 severity to better define public health policy and protect susceptible individuals, including specific social distancing measures. These measures can also contribute to preventing collapse of the health care system. Further research should define proper therapeutics for COVID-19, and nutritional therapy is an essential measure for treating COVID-19 in patients with obesity. Research should establish protocols, considering the severity of the disease.
